# An Atomistic Investigation of Cobalt’s Nanoindentation Response with An Angular Dependent Potential

**DOI:** 10.1021/acsomega.5c11093

**Published:** 2026-01-23

**Authors:** Douglas S. Oliveira, Danilo P. Kuritza, José E. Padilha, Mônica A. Cotta

**Affiliations:** † 28122 Universidade Federal do Paraná, Campus Avançado de Jandaia do Sul, 86900-000 Jandaia do Sul, Paraná, Brazil; ‡ Instituto de Física Gleb Wataghin, 28132 Universidade Estadual de Campinas, 13083-859 Campinas, São Paulo, Brazil

## Abstract

Cobalt and its alloys are essential in many advanced technologies and understanding their mechanical properties at the nanoscale is crucial for designing next-generation materials. In this work, an angular-dependent potential for cobalt was developed by fitting to a reference data set of atomic forces, energies, and stress tensors derived from first-principles density functional theory calculations. The potential’s performance was systematically evaluated against experimental data and two established classical potentials—an embedded-atom method potential and a modified embedded-atom method potential—across a range of structural, mechanical, thermal, and defect properties for both HCP and FCC phases, as well as the liquid state. The ADP model demonstrates a favorable balance between accuracy and computational cost, exhibiting a mean absolute percentage error of 6.3% for mechanical and elastic properties. Large-scale molecular dynamics simulations of nanoindentation on the (0001) basal plane of HCP cobalt were performed to investigate the atomistic mechanisms of plastic deformation. The simulations reveal that plasticity initiates with the nucleation of <a>-type dislocations on basal planes, followed by the activation of pyramidal <c+a> slip and a localized, reversible HCP-to-FCC phase transformation under high pressure. The critical shear stress for dislocation nucleation was found to decrease with increasing indenter radius, converging to a value of (13.7 ± 0.6) GPa.

## Graphical abstract

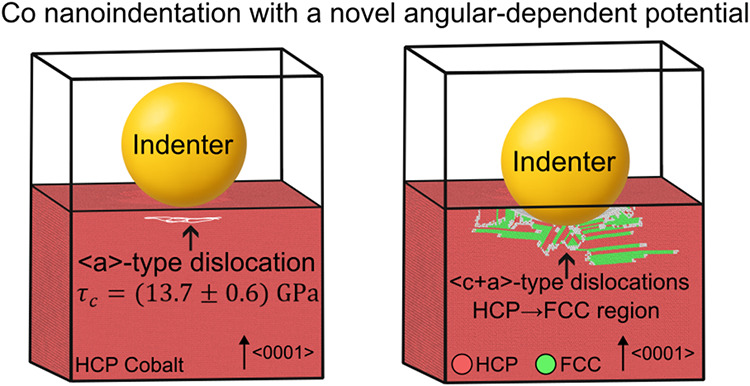

## 1 Introduction

Cobalt and its alloys are critical components in a wide array of advanced technologies, from high-strength superalloys used in aerospace turbines^1^ to high-capacity energy storage materials^2^ and biocompatible implants.^3^ The performance and reliability of these materials are intrinsically linked to their mechanical properties at the nanoscale, where fundamental mechanisms such as dislocation nucleation^4^ and phase transformations^5^ govern their response to stress. Understanding these atomistic processes is therefore essential for designing next-generation materials with enhanced durability and functionality.

Atomistic simulations provide an indispensable tool for investigating these phenomena, offering insights that are often inaccessible to direct experimental observation. While recent developments in machine-learning interatomic potentials (MLIPs) have enabled simulations with near first-principles accuracy, their substantial computational cost^6^ remains a barrier for the large-scale, mesoscale simulations required to model complex mechanical processes. Consequently, computationally efficient classical potentials that do not sacrifice physical fidelity remain highly attractive. A representative example of such multiscale challenges is the modeling of nanoindentation, a technique widely used to probe the mechanical strength of materials at the nanoscale. This process inherently links atomic-level mechanisms to macroscopic responses, making it a compelling test case for evaluating interatomic potentials. Accurate modeling of nanoindentation demands simulations involving millions of atoms in order to capture the collective evolution of extended defect structures that govern the plastic deformation behavior. Hence, achieving both computational efficiency and physical accuracy is essential for realistically reproducing such phenomena.

In this work, we explore the development of an angular-dependent potential (ADP)^7^ tailored for cobalt, aiming to balance accuracy and computational performance. The ADP framework builds upon the widely used embedded-atom method (EAM)^8^ by incorporating explicit angular-dependent terms, allowing a more refined description of atomic bonding without incurring the high costs of more complex approaches. We evaluate the proposed potential against a range of reference data and established models to assess its capabilities across key material properties.

To further evaluate the potential, we conduct large-scale simulations of nanoindentation in single-crystal HCP cobalt. These simulations are used to examine the onset of plasticity and the associated deformation mechanisms under indentation. Particular attention is given to the evolution of defect structures and stress-induced transformations, which are correlated with features observed in the load–displacement response. This analysis provides atomistic-level insights into the early stages of plastic deformation in cobalt.

## 2 Potential Development

We adopt an ADP framework, which builds upon the traditional EAM by incorporating angular dependencies into the atomic interaction model. Originally proposed by Mishin et al.,^7^ the ADP approach enhances the representation of interatomic forces by accounting for directional, noncentral interactions. The total energy within this model is formulated as

$$E_{\mathrm{tot}}=\frac{1}{2}\sum_{i,j\left(j\neq i\right)}\Phi_{\mathrm{ij}}\left(r_{\mathrm{ij}}\right)+\sum_{i}F_{i}\left({\mathrm{\rho ̅}}_{i}\right)+\frac{1}{2}\sum_{i,\alpha }{\left(\mu_{i}^{\alpha }\right)}^{2}+\frac{1}{2}\sum_{i,\alpha ,\beta }{\left(\lambda_{i}^{\alpha \beta }\right)}^{2}-\frac{1}{6}\sum_{i}\nu_{i}^{2}$$

Here, the angular contributions are defined as follows

$$\mu_{i}^{\alpha }=\sum_{j\neq i}u_{\mathrm{ij}}\left(r_{\mathrm{ij}}\right)r_{\mathrm{ij}}^{\alpha };\lambda_{i}^{\alpha \beta }=\sum_{j\neq i}w_{\mathrm{ij}}\left(r_{\mathrm{ij}}\right)r_{\mathrm{ij}}^{\alpha }r_{\mathrm{ij}}^{\beta };\nu_{i}=\sum_{\alpha }\lambda_{i}^{\alpha \alpha }$$

In these expressions, indices *i* and *j* represent atoms, and α,β = 1,2,3 correspond to Cartesian components. The function Φ_*ij*_(*r*_*ij*_) describes the short-range repulsive interaction between atoms, while $F_{i}\left(\overset{®}{\rho_{i}}\right)$ denotes the embedding energy based on the effective local electron density $\overset{®}{\rho_{i}}$ . The additional terms μ_*i*_^α^ and λ_*i*_^*αβ*^ introduce angular sensitivity into the potential, allowing for the modeling of directionally dependent interactions. The scalar ν_*i*_, derived from the trace of the λ tensor, summarizes the local structural anisotropy around atom *i*.

To parametrize the interatomic potential, we adopted a force-matching approach implemented via PotFit software.^9^ In this method, potential parameters are optimized by minimizing the deviation between predicted and reference quantities (atomic forces, total energies, and stress tensors) derived from first-principles calculations.

The reference data were generated using DFT as implemented in the VASP code,^10^ with the projector augmented-wave (PAW) method and the Perdew–Burke–Ernzerhof (PBE) exchange-correlation functional. A plane-wave energy cutoff of 350 eV was used, along with spin polarization. For periodic structures, a Γ-centered 3 × 3 × 3 k-point mesh was employed to sample the Brillouin zone.

The data set comprises 71 configurations of Co in HCP, FCC, liquid, and low-coordination environments. For both crystal structures, we included bulk cells under isotropic volumetric expansion/compression (±4 and ±8%) and uniaxial strains (±2 and ±6%) along directions associated with all independent elastic constants. Finite-temperature configurations spanning 200–2200 K were also considered. Additionally, the database contains slab geometries exposing the HCP (0001) and FCC (100)/(111) surfaces, single-vacancy configurations in both phases, a Co dimer, and liquid Co configurations at 2500 K.

Further details regarding the construction of the data set, as well as the final parameter set obtained for the angular-dependent potential, are provided in the Supporting Information (SI).

## 3 Interatomic Potential Framework and Simulation Methodology

To assess the performance of our interatomic potential, we computed key structural and energetic properties of cobalt in both HCP and FCC phases. The results were compared with available experimental data and two classical potentials: an EAM potential developed by Pun and Mishin,^11^ and the MEAM potential by Sharifi and Wick.^12^ Notably, the MEAM potential was designed to model a broader range of elements, including Cu, Ti, Ni, Cr, Co, Al, Fe, and Mn.

All molecular dynamics simulations used to evaluate structural and thermodynamic properties were performed with the LAMMPS software package,^13^ using a time step of 1 fs. Energy conservation tests, which verify the stability of this time step within the NVE ensemble, are presented in the SI. Additional details regarding the simulation protocols and property evaluation methods are also provided in the SI.

From a modeling standpoint, these families differ solely in how many-body metallic cohesion is encoded: EAM^8^ represents the total energy as a sum of pair terms plus an embedding functional of a scalar host electron density at each site, $E_{\mathrm{EAM}}=\frac{1}{2}\sum_{i,j\left(j\neq i\right)}\Phi_{\mathrm{ij}}\left(r_{\mathrm{ij}}\right)+\sum_{i}F_{i}\left({\mathrm{\rho ̅}}_{i}\right)$ . MEAM^14^ modifies EAM by decomposing *ρ̅*_*i*_ into angular partial densities and introducing screening functions, so the embedding depends on both magnitude and orientational content of the local environment. Meanwhile ADP keeps the EAM framework but adds explicit angular contributions—low-rank local moments constructed from bond directions—so the energy is *E*_*ADP*_ = *E*_*EAM*_ + *E*_*angular*_, providing orientational sensitivity without MEAM’s screening formalism.

Beyond these differences in mathematical formulation, two distinctions in practical implementation deserve particular attention. First, the EAM and MEAM potentials considered in this study represent interatomic interactions using analytic functions described by a fixed number of parameters—13 in the case of the EAM potential and 23 for the MEAM potential. In contrast, our model employs cubic spline functions, which are defined by the derivative values at the end points and a flexible number of internal knots. In this implementation, we use 52 free parameters. This flexibility can help the potential capture additional features, depending on the choice of training data and parametrization, though it may also increase the risk of overfitting if not carefully managed.

Second, our potential was optimized exclusively using DFT data, with the sole exception of the cohesive energy of HCP cobalt, which was used to adjust the reference energy of an isolated Co atom. On the other hand, the EAM potential incorporates several experimental values into its fitting process, including the lattice parameter, elastic constants, vacancy formation energy, and cohesive energy of HCP Co. The MEAM potential similarly relies on experimental elastic constants, cohesive energy, vacancy formation energy and nearest neighbor distance. While the inclusion of experimental data can improve agreement with specific properties, it may also introduce systematic discrepancies, as quantities such as elastic constants and lattice parameters are temperature dependent. These values are typically measured at room temperature, whereas the fitting procedures are conducted at 0 K.

## 4 Results and Discussion

Having established the methodological framework and potential formulations, we now present a comparative evaluation of the ADP, EAM,^11^ and MEAM^12^ potentials for cobalt in both HCP and FCC phases. A comprehensive summary of these results is presented in Tables 1 and 2.

**Table 1:** Physical Properties of Cobalt in the HCP Phase^a^,^b^

HCP				
property	experiment	ADP	EAM	MEAM
*a* (Å) (291 K)	2.5013 ^15^	2.4962*	2.5310*	2.5147*
c/a (291 K)	1.623 ^15^	1.633*	1.611*	1.622*
B(GPa) (300 K)	190 ^16^	193.5*	181.6*	180.6*
C11(GPa) (298 K)	307.1 ^17^	283.0*	289.8*	290.0*
C12(GPa) (298 K)	165 ^17^	159.8*	137.2*	130.3*
C13(GPa) (298 K)	102.7 ^17^	122.6*	114.7*	122.1*
C33(GPa) (298 K)	358.1 ^17^	358.6*	321.5*	303.9*
C44(GPa) (298 K)	75.5 ^17^	59.1*	80.3*	72.4*
*E*_c_ (eV/atom)	–4.39 ^18^	–4.385*	–4.391 ^11^	–4.404*
γ_*s*_ (0001) (mJ/m^2^)	2550 ^19^	2133*	2315 ^11^	1989*
γ_*I*_2__ (mJ/m^2^)	27 ^20^	44.6*	39.8 ^11^	69.4*
*E*_*v*_^*f*^ (eV)	1.4 ^19^	1.50*	1.49 ^11^	1.54*
*E*_*v*_^*m*^ (eV)	-	0.99*	0.98 ^11^	0.85*
*T* ^*hcp → fcc*^ (K)	695 ^21^	-	717 ^11^	-
Δ*H* ^*fcc → hcp*^ (eV)	–0.0044 ^21^	–0.0084*	–0.0061 ^11^	–0.01195*
Cv (300 K) J/K/mol	24.73 ^21^	23.12*	22.51*	24.94*

a Experimental values are shown alongside results obtained in this work using ADP, EAM, and MEAM potentials.

b Values marked with * were calculated in this work.

**Table 2:** Physical Properties of Cobalt in the FCC Phase^a^,^b^

FCC				
property	experiment	ADP	EAM	MEAM
*a* (Å) (291 K)	3.5369 ^15^	3.5179*	3.5807*	3.5492*
B(GPa) (300 K)	182 ^22^	182.7*	190.9*	179.77*
C11(GPa) (298 K)	225 ^22^	210.0*	262.9*	249.7*
C12(GPa) (298 K)	160 ^22^	166.0*	152.8*	144.3*
C44(GPa) (298 K)	92 ^22^	106.9*	99.4*	94.8*
*E*_c_ (eV/atom)	-	–4.377*	–4.3849	–4.3927*
γ_*s*_(100)(mJ/m^2^)	-	2440*	2470 ^11^	2163*
γ_*s*_(110)(mJ/m^2^)	-	2584*	2604 ^11^	2103*
γ_*s*_(111)(mJ/m^2^)	-	2172*	2333 ^11^	1943*
*E*_*v*_^*f*^ (eV)	1.34 ^23^	1.6*	1.56 ^11^	1.51*
*E*_*v*_^*m*^ (eV)	-	0.94*	0.95 ^11^	0.87*
Cv (300 K) J/K/mol	24.73 ^21^	23.12*	22.51 ^11^	24.94*
*T*_m_ (K)	1768 ^21^	1550*	1898 ^11^	1560*

a Experimental values are shown alongside results obtained in this work using ADP, EAM, and MEAM potentials.

b Values marked with * were calculated in this work.

The ADP potential exhibits high fidelity in predicting the mechanical and elastic properties of cobalt, achieving a mean absolute percentage error (MAPE) of 6.3%, compared to 7.1% for EAM and 7.4% for MEAM. Notably, all three potentials accurately reproduce the experimental cohesive energy (E_c_) of HCP cobalt, which is expected given that this quantity was explicitly included in their respective fitting procedures.

The performance of the potentials was also evaluated for surface and defect-related properties. The ADP potential underestimates the (0001) surface energy (γ_*s*_) of HCP cobalt and significantly overestimates the intrinsic stacking-fault energy (γ_*I*_2__), a trend also observed with the MEAM potential. For FCC cobalt, experimental surface energy values are unavailable; however, both ADP and EAM correctly reproduce the expected energetic hierarchy among low-index surfaces (γ_*s*_(110) > γ_*s*_(100) > γ_*s*_(111)^24^), whereas the MEAM potential fails to capture this trend. Regarding point defects, all three models yield reasonable estimates for the vacancy formation energy (*E*_*v*_^*f*^) in the HCP phase but systematically overestimate this property for the FCC phase relative to experimental data. In addition, the vacancy migration energies (*E*_*v*_^*m*^) predicted by the models are reported in Tables 1–2 and provide the complementary input needed for vacancy-mediated diffusion analyses. The computed lattice heat capacity at constant volume (Cv) shows good agreement with experimental values across all potentials.

All three models correctly predict a negative enthalpy difference between the HCP and FCC phases at 0 K (Δ*H* ^*fcc* → *hcp*^), in agreement with experimental observations. However, they all overestimate the magnitude relative to the experimental value (−0.0044 eV/atom). MEAM (−0.01195 eV/atom) and ADP (−0.0084 eV/atom) exhibit the largest overbinding, while EAM (−0.0061 eV/atom) is closest to experiment. This systematic overbinding shifts the HCP → FCC transition (*T* ^*hcp* → *fcc*^) to higher temperatures;^25^ the larger bias in ADP and MEAM likely explains why the transition is not observed in simulations with these models, whereas the smaller EAM error still allows the transition to be captured.

Regarding the transition to the liquid phase, for the melting temperature (*T*_m_) of FCC cobalt, the ADP model predicts 1550 K, underestimating the experimental value by approximately 220 K—a result similar to that of the MEAM potential (1560 K). The EAM model, on the other hand, overestimates the melting point by about 130 K. Properties of the liquid state were also examined, and Figure 1 compares the predicted density of liquid cobalt against experimental data. In the low-temperature regime, near the experimental melting point (∼1800 K), both the ADP and MEAM potentials yield density values that fall within the experimental uncertainty range reported by Assael et al.^26^ At higher temperatures (>2200 K), however, the EAM potential shows a better agreement with the experimental trend.

**Figure 1:**
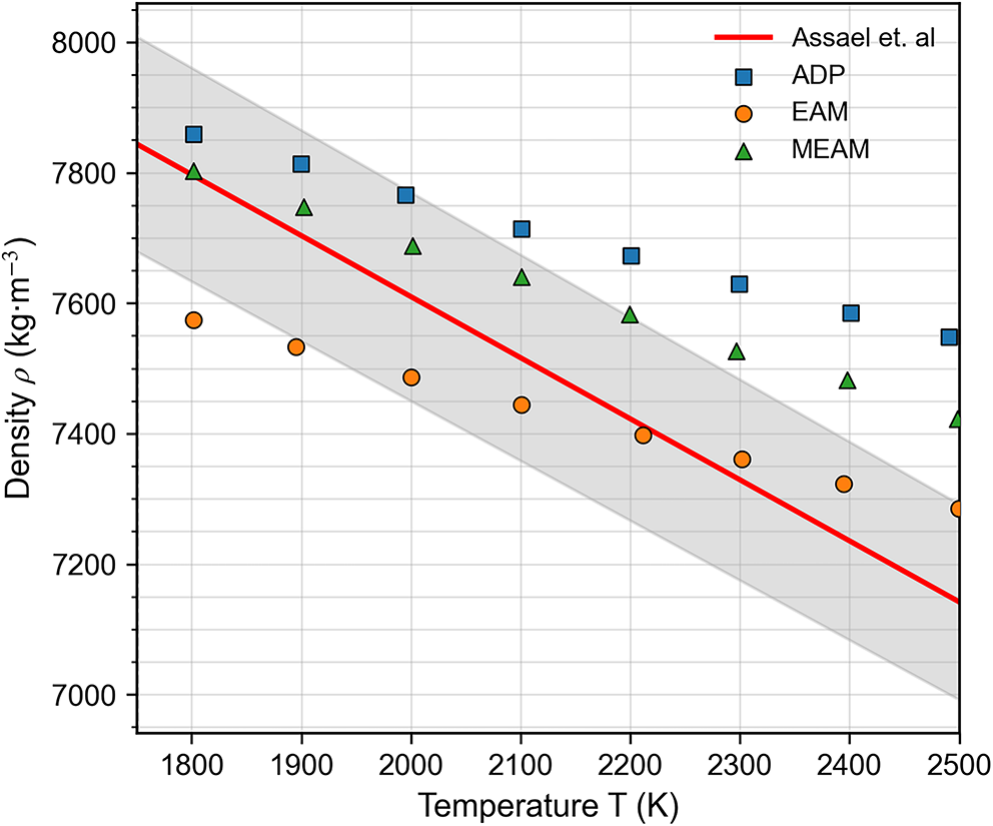
Temperature dependence of the liquid cobalt density as predicted by the ADP, MEAM, and EAM potentials, compared against experimental data compiled by Assael et al.^26^ The shaded band indicates ±2σ (95% confidence level) for the Assael et al. reference correlation.

As for viscosity, Figure 2 presents the predicted values for liquid cobalt. The ADP and MEAM potentials tend to underestimate viscosity at lower temperatures (∼1800 K), but their predictions fall within the experimental range^26^ at higher temperatures (∼2100 K). In contrast, the EAM potential overestimates viscosity at elevated temperatures while aligning better with experimental values near the melting point.

**Figure 2:**
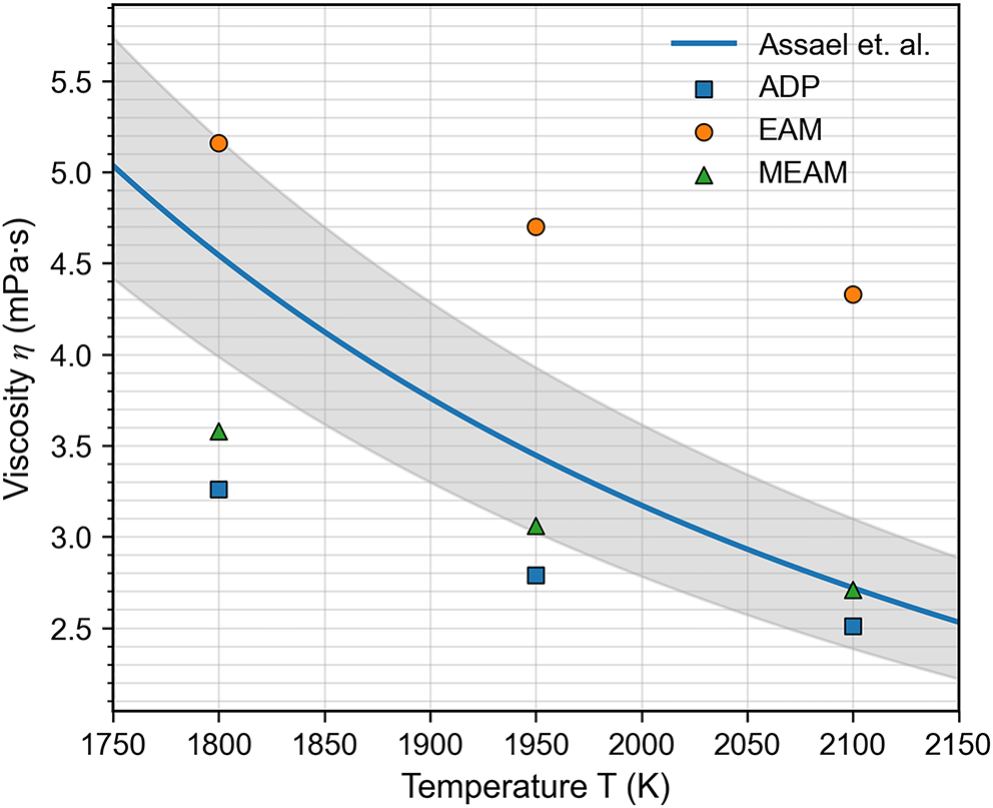
Temperature dependence of the liquid cobalt viscosity as predicted by the ADP, MEAM, and EAM potentials, compared against experimental data compiled by Assael et al.^26^ The shaded band indicates ± 2σ (95% confidence level) for the Assael et al. reference correlation.

The phonon dispersion relations, shown in Figures 3 and 4, further highlight the strengths of the ADP model. Both the ADP and MEAM potentials show excellent agreement with experimental phonon spectra for FCC and HCP phases. The EAM potential, however, tends to overestimate the phonon frequencies, particularly for the upper acoustic branches in the FCC phase and the optical branches in the HCP phase.

**Figure 3:**
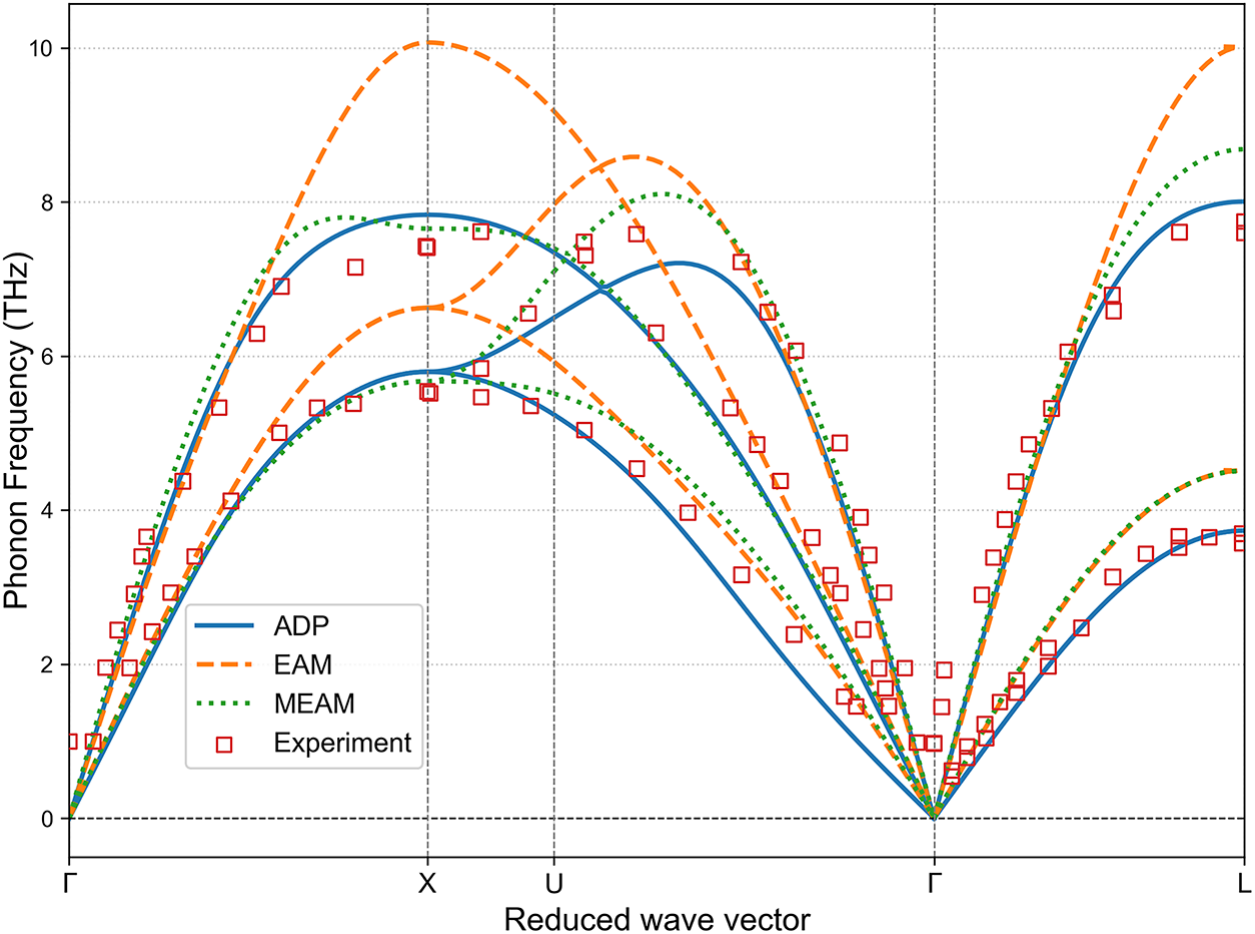
Phonon dispersion relations of cobalt in the FCC phase calculated using the ADP, EAM, and MEAM potentials. Experimental data from Strauss et al.^27^ are included for comparison.

**Figure 4:**
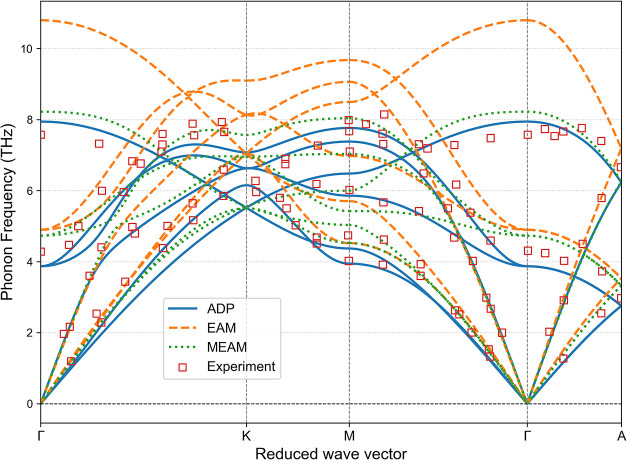
Phonon dispersion relations of cobalt in the HCP phase calculated using the ADP, EAM, and MEAM potentials. Experimental data from Wakabayashi et al.^28^ are included for comparison.

The linear thermal expansion is presented in Figure 5. All three interatomic potentials produce values that are in reasonable agreement with the experimental data available in the literature. Among them, the EAM potential shows the closest match to the reference values over the entire temperature range.

**Figure 5:**
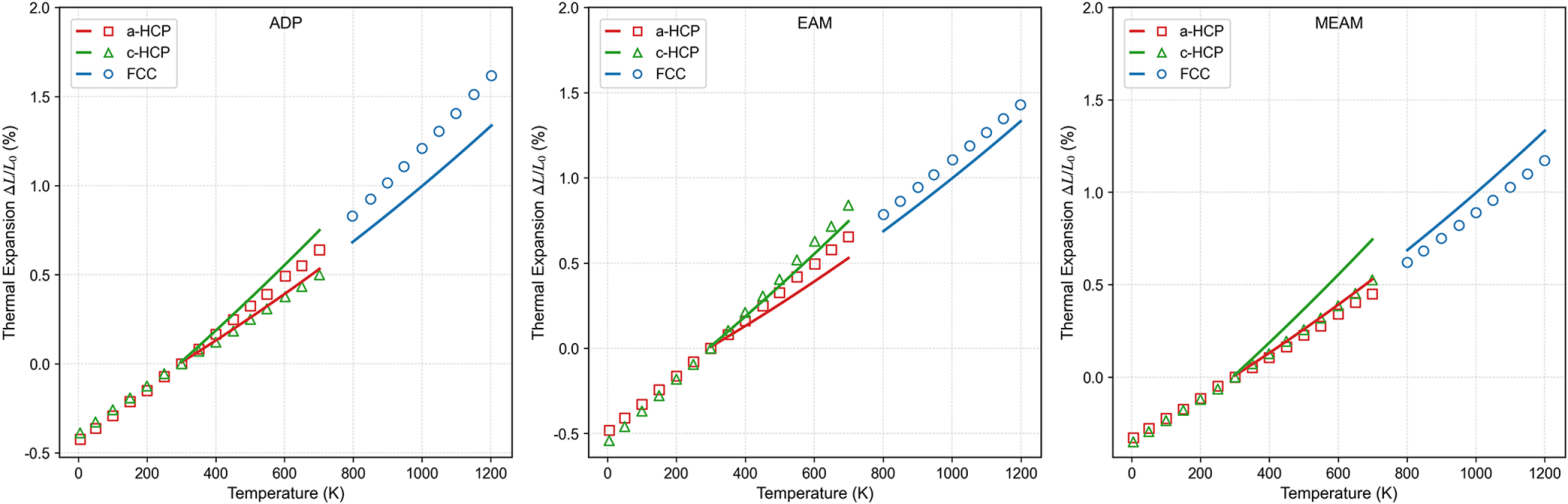
Linear thermal expansion as a function of temperature for the ADP, EAM, and MEAM potentials, relative to the value at 300 K. Solid lines represent experimental data from Touloukian et al.,^29^ while symbols correspond to the values obtained from each potential.

Finally, from a practical perspective, the ADP potential represents a reasonable compromise between accuracy and computational cost. As shown in Table 3, the EAM potential is approximately twice as fast as ADP, with a normalized relative time of 0.47, while MEAM is nearly four times more computationally expensive, with a value of 3.92. Compared to EAM, ADP offers improved accuracy in several key areas, particularly when vibrational and transport properties are of interest. It exhibits the lowest mean absolute percentage error for elastic constants, provides a closer match to experimental phonon dispersions in both FCC and HCP phases, and maintains liquid viscosities within experimental ranges at high temperatures, where EAM tends to overestimate. These characteristics make ADP a suitable choice for large-scale molecular dynamics simulations that require a balance between fidelity and efficiency.

**Table 3:** Relative Computational Cost of Interatomic Potentials, Normalized to the ADP Potential

potential	ADP	EAM	MEAM
normalized relative time	1.00	0.47	3.92

### 4.1 Nanoindentation in HCP Cobalt

We applied our newly developed interatomic potential to investigate the atomistic mechanisms governing nanoindentation in HCP cobalt, with particular focus on dislocation nucleation. Simulations were carried out at room temperature (300 K), with indentation performed normal to the (0001) basal plane.

Prior to indentation, the system was equilibrated to eliminate residual stresses and accommodate thermal expansion. This equilibration was performed under the isothermal–isobaric (NPT) ensemble at 300 K and zero external pressure for 10 ps, allowing the simulation cell to deform freely in all three spatial directions. Although periodic boundary conditions were applied in every dimension—leading some atoms to reappear at the top boundary—these atoms had no physical interaction with the free surface and thus did not influence the indentation response.

A schematic illustration of the simulation setup, including the indenter geometry and boundary conditions, is shown in Figure 6.

**Figure 6:**
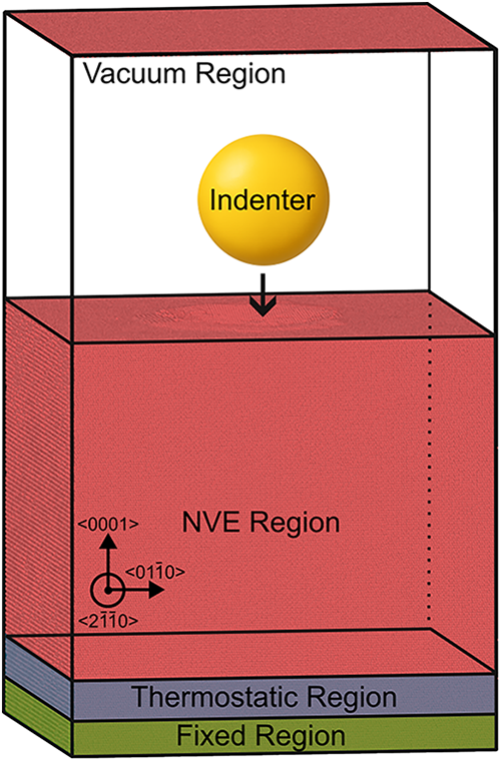
Schematic representation of the molecular dynamics nanoindentation setup for HCP cobalt. The spherical indenter is positioned above the (0001) basal plane, with indentation applied along the [0001] direction (*Z*-axis). Periodic boundary conditions are imposed in all directions, and the simulation cell includes fixed layers at the bottom to mimic bulk constraints, a thermostatic region governed by a Langevin thermostat, and a free surface to allow realistic deformation behavior.

After equilibration, the bottom seven atomic layers of the crystal were fixed to mimic bulk mechanical constraints, while the adjacent seven layers were treated as a thermostatic region. In this region, a Langevin thermostat was employed within a microcanonical (NVE) ensemble to emulate heat dissipation effects caused by the indentation process.^30^ The remainder of the system evolved in a pure NVE ensemble, allowing for unimpeded energy transfer and realistic defect dynamics.

Unless otherwise specified, all simulations were conducted using a simulation cell of approximately 36 nm × 36 nm × 35 nm, containing ∼4.19 million atoms, and a spherical indenter moving at a constant velocity of 20 m/s. The [0001] crystallographic axis was aligned with the *Z*-direction. To verify that the results were statistically robust and not an artifact of the initial atomic velocities, key simulations were repeated using different random seeds.

One of the main challenges in nanoindentation simulations is managing size effects. Parameters such as indenter radius, indentation velocity, and the overall dimensions of the simulation domain can significantly affect the mechanical response and the mechanisms of defect nucleation.^31^ To address these concerns, we conducted a systematic investigation of dislocation nucleation as a function of indenter size.

Special attention was given to ensuring that the simulation domain was large enough to capture the early stages of plasticity without interference from periodic boundaries. We focused particularly on the nucleation of the first dislocation, which typically coincides with the initial load drop, or “pop-in” event. In our simulations, this occurred at indentation depths below 1 nm. For the largest indenter radius used (16 nm), the corresponding contact diameter at that depth was approximately 11 nm. Even under these conditions, dislocation loops remained fully contained within the simulation box, indicating that boundary effects did not distort the observed mechanisms.

In our nanoindentation simulations of HCP cobalt along the (0001) axis, dislocation extraction analysis (DXA) and common neighbor analysis (CNA), both performed using OVITO,^32^ reveal key aspects of the deformation mechanisms. DXA shows that plastic deformation initiates via the nucleation of <a>-type dislocations, which expand as planar loops on the basal (0001) plane beneath the indenter (Figure 7a). This behavior is consistent with TEM observations, which indicate that basal slip is the initial mode of plastic deformation activated in HCP cobalt.^33^ As indentation depth increases, the defect structure evolves, and nonbasal plasticity is activated. We observe the formation of dislocation segments with a *c*-axis component, indicative of the activation of pyramidal ⟨c+a⟩ slip systems (Figure 7b).

**Figure 7:**
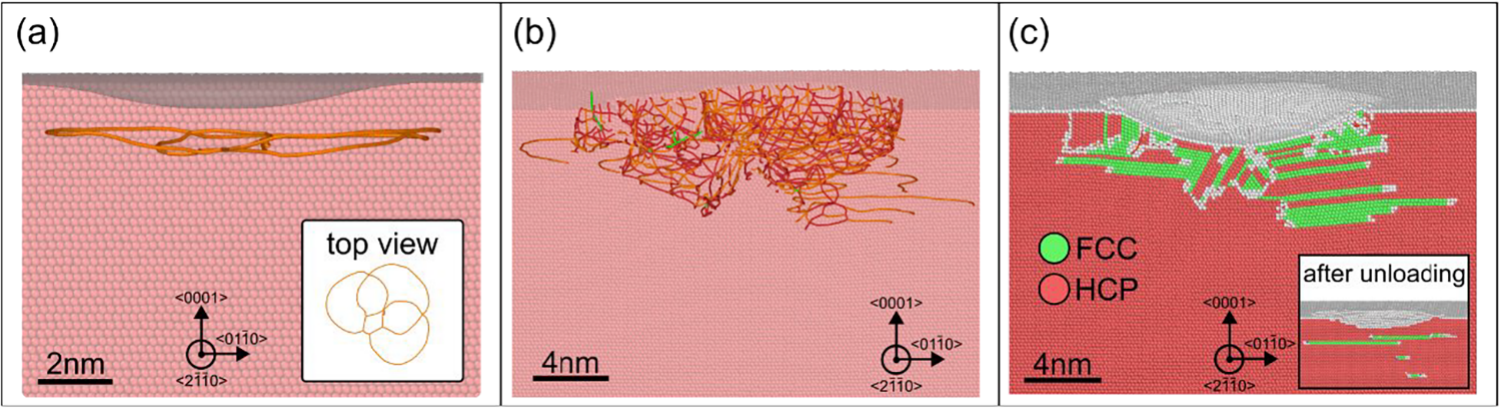
(a) Side view and top view (inset) of an HCP Co crystal shortly after the nucleation of the first dislocations. Orange lines indicate dislocations with Burgers vector <1/3⟨1–100⟩>, associated with basal slip. The gray area at the top of (a) represents the surface sink-in caused by the indenter. (b) and (c) Side view of a slice of the crystal at an indentation depth of 2 nm, showing the evolution of the defect structure with activation of dislocations containing a *c*-axis component and the emergence of FCC regions (in green). The inset in (c) shows the structure after unloading, highlighting partial reversal of the FCC phase back to HCP.

CNA reveals that at larger penetration depths, local stacking transitions from HCP to FCC occur beneath the contact zone, as shown in Figure 7c. Given the small free energy difference between the two phases and the intense hydrostatic compression during indentation, this transformation (ABAB → ABC stacking) can occur under high pressure.^34^ Upon unloading, the release of pressure and shear allows a partial reverse FCC → HCP transformation (Figure 7c, inset), consistent with the reversible martensitic nature of the FCC ↔ HCP transition in cobalt and with the thermodynamic preference of the HCP phase at ambient conditions.^25^

To further quantify the onset of plasticity, we estimated the critical shear stress for dislocation nucleation (τ_c_). Based on classical contact mechanics theory,^35^ τ_c_ can be inferred from the indentation load (*P*) and depth (*h*) at the first pop-in event. For a spherical indenter, the surface contact pressure (*p*_0_) is expressed as

1 $$p_{0}=\frac{3P}{2\pi a^{2}}$$

Assuming purely elastic Hertzian contact, the contact radius (*a*_*c*_) can be related to the indenter radius (*R*) and indentation depth by

2 $$a_{c}=\sqrt{Rh}$$

Substituting this into the expression for *p*_0_, we obtain

3 $$p_{0}=\frac{3P}{2\pi \mathrm{Rh}}$$

The maximum shear stress occurs beneath the surface at a depth of approximately 0.48*a*_*c*_ and can be estimated as τ_*max*_ ≈ 0.31*p*_0_ for Poisson’s ratio ν = 0.30 (the prefactor 0.31 varies slightly with Poisson’s ratio^36^).

At the first pop-in event, dislocation nucleation occurs, and the maximum shear stress is assumed to reach the critical value τ_*c*_. Therefore, using the indentation depth at pop-in *h*_*pop‑in*_, we estimate

4 $$\tau_{c}=0.31\frac{3P_{\mathrm{pop}‐n}}{2\pi Rh_{\mathrm{pop}‐\mathrm{in}}}$$

This analytical formulation provides a convenient means of estimating the critical shear stress for dislocation nucleation directly from the load versus displacement (*P* versus *h*) curves obtained in nanoindentation simulations. As shown in Figure 8, the initial portion of these curves corresponds to the elastic response and varies with the indenter radius. This variation influences both the onset and the magnitude of the first pop-in event, reflecting the dependence of the critical load for plasticity initiation on indenter size.

**Figure 8:**
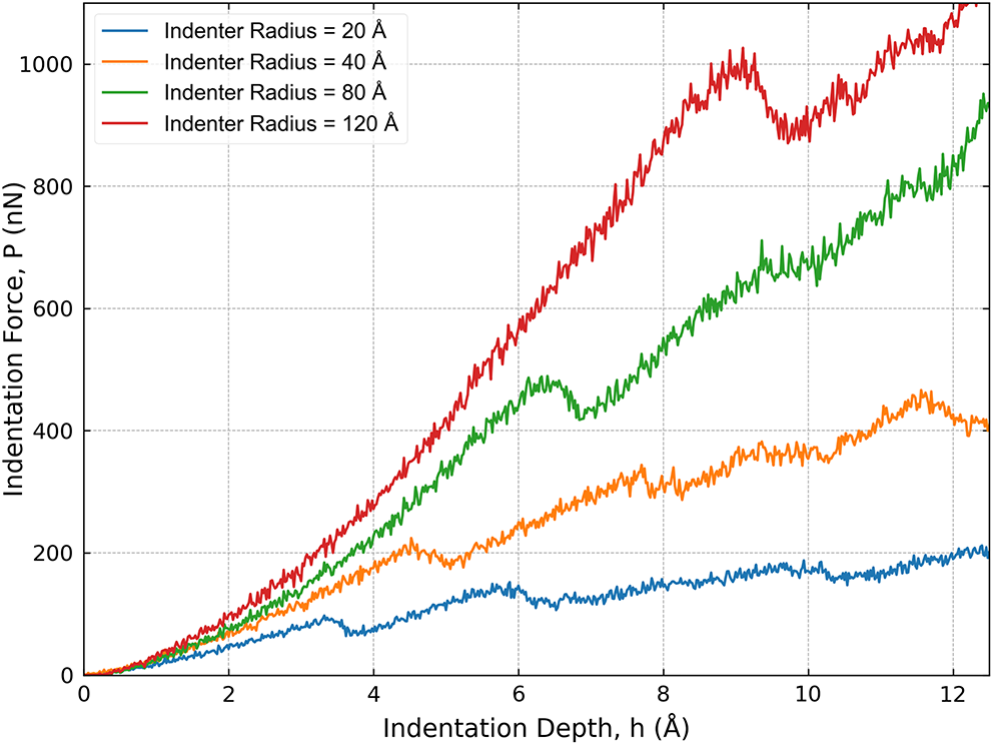
Load–displacement (P–h) curves for spherical indenters of varying radii.

Using the values of *P*_*pop‑in*_ (with an estimated uncertainty of 10%) and *h*_*pop‑in*_ (with an estimated error of 0.5 Å), extracted from the load versus displacement curves, we calculated the τ_*c*_ for each simulation. The resulting values are presented in Figure 9.

**Figure 9:**
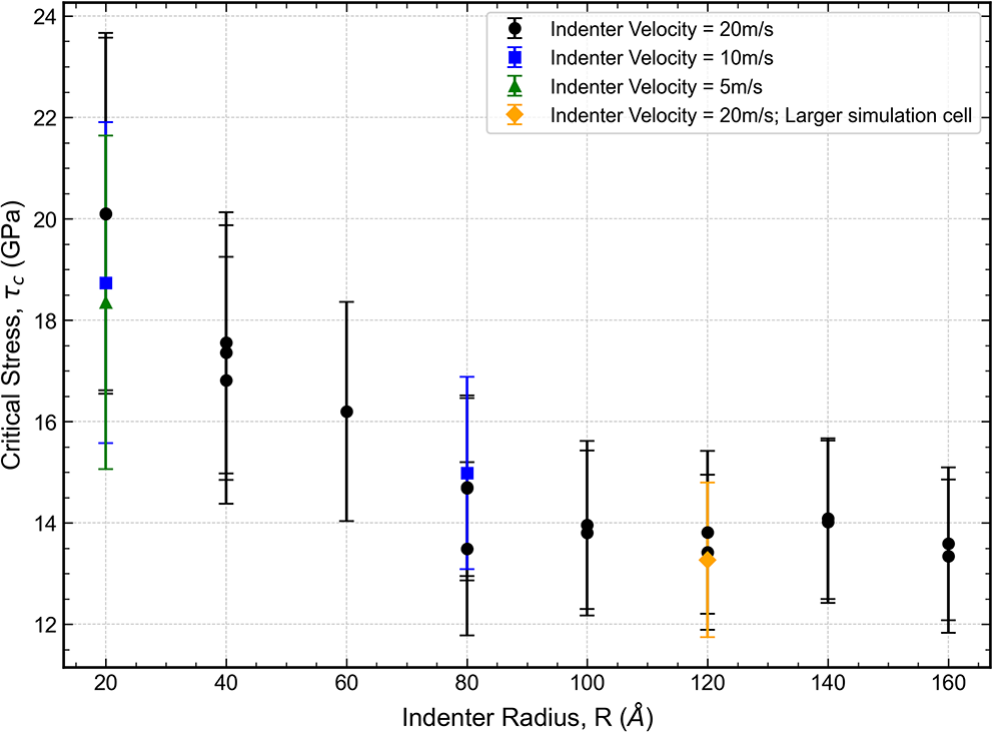
Estimated critical shear stress τ_*c*_ for dislocation nucleation as a function of indenter radius, computed from the pop-in load *P*_*pop‑in*_ and indentation depth *h*_*pop‑in*_.

To ensure that the results are not affected by the choice of indentation velocity, we included additional simulations with lower indenter velocities of 10 and 5 m/s. Furthermore, to verify that finite size effects did not influence the results, we performed an additional simulation using a larger simulation cell measuring 60.2 nm × 60.3 nm × 34.9 nm, containing a total of 11,722,480 atoms.

The estimated critical shear stress, τ_*c*_, was observed to be inversely dependent on the spherical indenter radius, asymptotically approaching a constant value as the radius increases. This trend is distinct from the indentation size effect associated with dislocation pop-in reported by Shim et al.,^37^ whose “smaller is stronger” behavior was measured with significantly larger tip radii (from ∼0.58 μm to ≥17.5 μm). Instead, our findings align with the near-surface dislocation nucleation model proposed by Kelchner et al.^38^ In this framework, when contact dimensions are near-atomistic, the stressed volume becomes comparable to the dislocation line width. As a result, surface and image forces elevate the nucleation barrier, making the average shear stress across the nascent dislocation loop—rather than the peak stress—the relevant criterion for yielding. For indenter radii greater than or equal to 100 Å, the values of τ_*c*_ become relatively stable, yielding an average of (13.7 ± 0.6)­GPa.

First-principles generalized stacking-fault energy (GSFE) calculations for hcp Co predict an ideal shear strength on the basal plane of ≈6.95 GPa.^39^ A classical upper-bound (Frenkel) estimate, $\tau_{\mathrm{ideal}}\approx \frac{\mu }{2\pi }$ , using the basal shear modulus μ = C_66_ = (C_11_–C_12_)/2 ≈ 71 GPa at room temperature,^17^ gives τ_*ideal*_ ≈ 11.3 GPa. Our value therefore exceeds the DFT ideal by roughly a factor of 2 and the Frenkel estimate by ∼20%, which is consistent with the elevation of the shear stress required for dislocation nucleation under the strong triaxial compression characteristic of indentation compared with the relaxed ideal shear of a perfect crystal.^40^ Consequently, this critical stress corresponds to the theoretical strength limit probed during experimental ’pop-in’ events in defect-free volumes, and is significantly higher than experimental macroscopic hardness values (typically 2–3 GPa for bulk cobalt^41^) which are governed by the motion of pre-existing defects. Recent studies have highlighted the sensitivity of this elastic-to-plastic transition to local structure. For instance, Wang et al.^42^ demonstrated that in Fe-based amorphous alloys, pop-in events are governed by the activation of shear transformation zones, whereas in our crystalline HCP Co, the mechanism is strictly dislocation nucleation. Furthermore, consistent with recent experimentally validated MD studies of cemented carbides where indentation pressure triggered FCC → BCC transformations in the binder phase,^43^ our simulations reveal that the hydrostatic pressure beneath the tip is similarly sufficient to drive a local HCP → FCC transformation in pure Cobalt. Importantly, these GPa-level τ_*c*_ metrics are not directly comparable to macroscopic critical resolved shear stresses (CRSS) for basal slip in cobalt (≈7–11 MPa at room temperature), which quantify the stress to move pre-existing dislocations rather than to nucleate them.^44^

From the estimated value of τ_*c*_ and the *h*_*popin*_ versus R trend, the reduced modulus E* can be extracted by rearranging classical contact mechanics expressions.^35^ The applied load during elastic spherical indentation is related to the contact radius and indenter radius by

5 $$P=\frac{4}{3}E^{*}\frac{{a_{c}}^{3}}{R}$$

By substituting eqs 2 into [Disp-formula eq5] and combining the result with eq 4, we obtain an explicit expression for the indentation depth at the onset of plasticity

6 $$h_{\mathrm{pop}‐\mathrm{in}}={\left(\frac{\tau_{c}}{0.197E^{*}}\right)}^{2}R$$

Figure 10 shows the relationship between *h*_*popin*_ and the indenter radius *R*, along with the corresponding linear regression. The slope of this linear fit enables the determination of the reduced modulus E* according to eq 6.

**Figure 10:**
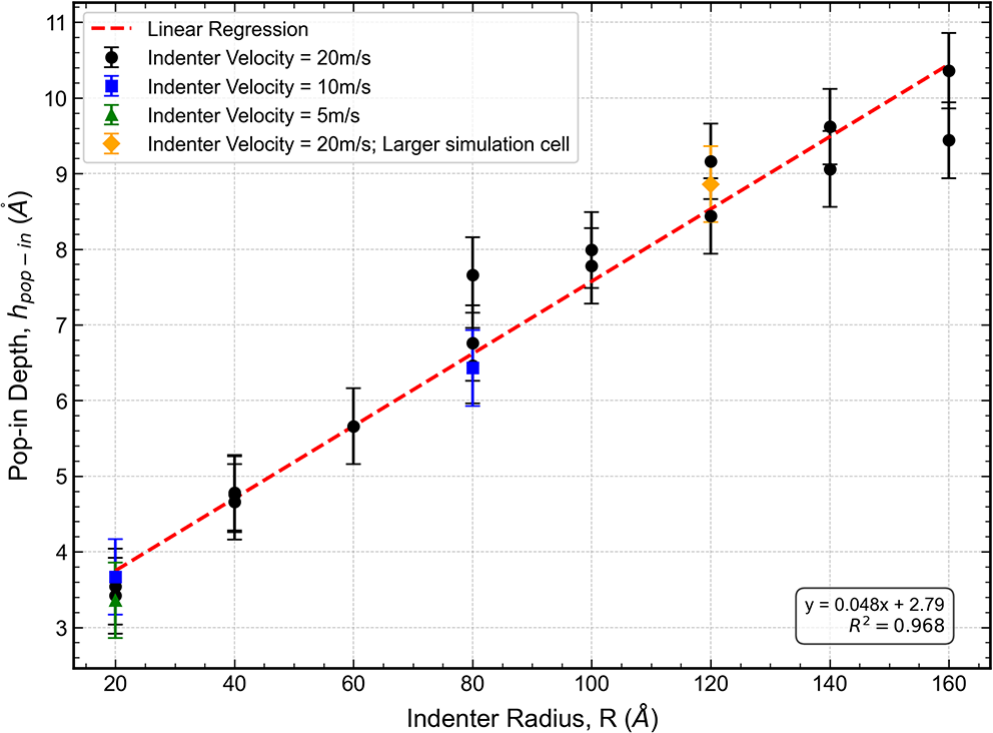
Onset indentation depth *h_pop‑in_* vs indenter radius *R*, showing a linear trend consistent with eq 6. The slope allows extraction of the reduced modulus E*, using the estimated shear stress τ_*c*_.

Using the slope of the linear fit in the plot of *h*_*popin*_ versus R, together with the estimated critical shear stress τ_*c*_ = (13.7 ± 0.6)­GPa, we determined the reduced modulus E* = (317 ± 15)­GPa. Applying a Poisson’s ratio of v_31_ = 0.277, obtained from the ADP potential’s elastic constants (see Table 1), yields a corresponding Young’s modulus of *E* = (293 ± 14)­GPa which is close to a previously calculated value of 313 GPa, reported for similar systems.^45^

An alternative method for determining E* involves directly fitting the elastic portion of the load–displacement curve—prior to the first pop-in—using Hertzian contact theory, which describes the relationship as

$$P=\frac{4}{3}E^{*}\sqrt{R}h^{3/2}$$

We noted that the value obtained from this fit is sensitive to the specific data range selected from the loading curve. To ensure a robust estimation, we adopted a systematic procedure: numerous fits were performed for each simulation while systematically varying the start and end points of the fitting interval. The final value was calculated as the average of the results from the top 10% of fits, ranked by the coefficient of determination (*R* ^2^). The associated uncertainty was taken as the standard deviation of this same subset of fits. The high quality of the selected fits, which typically exhibited *R* ^2^ > 0.99, confirms that the initial elastic response of the material is well-described by the Hertzian model. Furthermore, the clear demarcation between this Hertzian regime and the subsequent load drop demonstrates the potential’s ability to accurately resolve the transition from elastic deformation to discrete plasticity (pop-in). Figure 11 shows the value of E* as a function of indenter radius obtained through this method.

**Figure 11:**
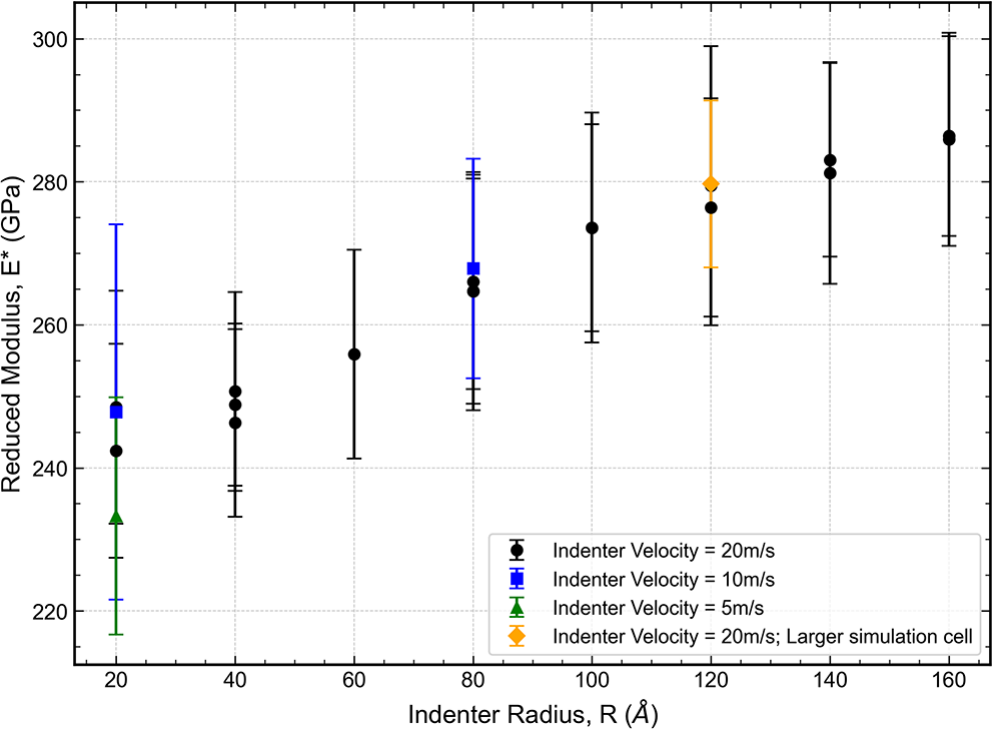
Reduced modulus E* vs indenter radius, obtained from Hertzian fits to the elastic loading curves. Values are averaged from top 10% fits by *R* ^2^.

The E* obtained in this way is strongly dependent on the assumed tip radius *R*, and still falls below the value of (317 ± 15)­GPa reported above. This slow convergence is expected because elasticity is long-range and fundamentally a bulk property. Consequently, when the tip radius is only a few atomic spacings, surface stress/energy and the limited number of atoms involved in the contact introduce pronounced finite-size and surface effects.^46,47^

In contrast, the onset of plasticity (e.g., dislocation nucleation) is governed by local, atomic-scale events.^48^ Accordingly, the intrinsic activation parameters of incipient plasticity (e.g., activation volume and enthalpy) do not require large indenters to be determined. It is worth noting, however, that macroscopic observables like pop-in loads or apparent flow stresses may still depend on contact size, due to sampling statistics over the stressed volume.

## 5 Conclusion

In this work, we have developed an angular-dependent potential for cobalt, constructed through a force-matching procedure based on first-principles DFT data. The potential was evaluated across a broad range of structural, mechanical, thermal, and dynamical properties for both HCP and FCC phases, as well as the liquid state. The ADP model shows consistent performance, yielding a mean absolute percentage error of 6.3% for mechanical and elastic properties—slightly lower than those observed for the EAM (7.1%) and MEAM (7.4%) potentials used for comparison. It also captures key features such as phonon dispersion relations and liquid-state properties near the melting point with reasonable agreement to experimental observations. In terms of computational efficiency, the ADP offers a favorable compromise between accuracy and cost, making it a practical choice for large-scale atomistic simulations.

The potential’s applicability was further explored through simulations of nanoindentation in HCP cobalt. The load–displacement response exhibited an initial elastic regime consistent with Hertzian contact mechanics, and the estimated Young’s modulus aligns with previously reported values from atomistic studies. The onset of plasticity, marked by the first pop-in event, is associated with the nucleation of ⟨a⟩-type dislocations on basal (0001) planes. As indentation progresses, deformation involves activation of pyramidal ⟨c+a⟩ slip systems to accommodate strain along the *c*-axis. Additionally, under high triaxial stress, a localized and reversible HCP-to-FCC transformation is observed beneath the indenter. Analysis of the critical shear stress for dislocation nucleation reveals a decreasing trend with increasing indenter size, approaching a limiting value of (13.7 ± 0.6) GPa. These findings provide a coherent link between atomistic mechanisms and macroscopic mechanical behavior.

Regarding transferability and limitations, the potential exhibits robust stability under nonequilibrium conditions typical of atomistic simulations. It maintains numerical stability and consistent physical behavior in the liquid state at temperatures up to at least 2500 K and captures high-pressure phase transformations (HCP → FCC) during high-strain-rate nanoindentation without numerical artifacts. However, users should be aware of specific physical limits. First, the potential overestimates the HCP→FCC enthalpy difference (as detailed in Section 4), which may shift the thermally induced solid-state phase transition to temperatures higher than experimental values. Second, as a classical model, it does not account for electronic excitations, ionization, or chemical reactivity.

In summary, the proposed ADP offers a physically sound and computationally efficient framework for simulating cobalt systems. Its overall agreement with experimental and theoretical benchmarks—along with its capacity to capture relevant defect mechanisms—suggests that it can be a valuable tool for studies involving mechanical deformation, phase transformations, and defect dynamics in pure cobalt. As cobalt is a fundamental component of many high-performance alloys, this potential serves as a robust foundation for future extensions to multielement systems. Given the modular nature of the ADP formalism, such extensions can be achieved by parametrizing only the cross-species interaction terms against binary reference data, while rigorously preserving the validated pure-element description established in this work. This approach enables the atomistic investigation of technologically critical materials like superalloys. Furthermore, while this classical model does not include explicit spin degrees of freedom, it implicitly incorporates magnetic effects through the use of spin-polarized DFT training data.

## Supplementary Material



## Notes

Details on the DFT reference data set; tabular data for ADP potential parameters (Tables S1–S5); extended methodology for potential characterization including crystal structure, cohesive energy, interfaces, stacking faults, point defects, phase transitions, phonons, and liquid state properties; numerical stability and energy conservation analysis (Figure S1) (PDF)
