# Supplementary material for: An Atomistic Investigation of Cobalt’s Nanoindentation Response with An Angular Dependent Potential
Source: ACS Omega. 2026 Jan 23;11(4):6324–33. doi: 10.1021/acsomega.5c11093 (PMC12878731; doi:10.1021/acsomega.5c11093)
Supplement: Supplementary file 1 [file ao5c11093_si_001.pdf]

An Atomistic Investigation of Cobalt's Nanoindentation Response with an Angular Dependent Potential

Douglas S. Oliveira*[1]; Danilo P. Kuritza[1]; José E. Padilha[1]; Mônica A. Cotta[2]

[1]Universidade Federal do Paraná, Campus Avançado de Jandaia do Sul, 86900-000 Jandaia do Sul, Paraná, Brazil

[2]Instituto de Física Gleb Wataghin, Universidade Estadual de Campinas, 13083-859 Campinas, São Paulo, Brazil

*Corresponding author: douglas.oliveira@ufpr.br

1. DFT Reference Dataset

The dataset comprises a total of 71 density functional theory simulations involving cobalt (Co) in various crystal structures and configurations:

- Face-Centered Cubic (FCC) Co Structures:
  - 4 simulations with 216 atoms each, with isotropic volumetric expansion and contraction of ±4% and ±8%.
  - 12 simulations with 216 atoms each, with uniaxial strain applied along directions corresponding to elastic constants C_{11}, C_{12}, and C_{44}, at ±2% and ±6%.
  - 1 simulation with 64 atoms exposing the (100) surface facet in contact with vacuum.
  - 1 simulation with 120 atoms exposing the (111) surface facet in contact with vacuum.
  - 1 fully relaxed structure with 216 atoms.
  - 11 simulations with 192 atoms each, sampled across temperatures ranging from 200 K to 2200 K.
  - 1 simulation with a single vacancy (215 atoms total).
- Hexagonal Close-Packed (HCP) Co Structures:
  - 4 simulations with 250 atoms each, with isotropic volumetric expansion and contraction of ±4% and ±8%.
  - 20 simulations with 250 atoms each, with uniaxial strain applied along directions corresponding to elastic constants C_{11}, C_{12}, C_{13}, C_{33}, and C_{44}, at ±2% and ±6%.
  - 1 simulation with 120 atoms exposing the (0001) surface facet in contact with vacuum.
  - 1 fully relaxed structure with 250 atoms.

- 11 simulations with 180 atoms each, sampled across temperatures ranging from 200 K to 2200 K.
- 1 simulation with a single vacancy (249 atoms total).

- Other Configurations:
  - 1 simulation with 2 cobalt atoms separated by a distance of 1.8 Å.
  - 3 simulations of liquid Co with 172 atoms at 2500 K.

To ensure alignment with experimental results, the energy offset of an isolated cobalt atom was chosen such that the computed cohesive energy matches the experimental cohesive energy of HCP Co.

2. ADP potential parameters

The ADP potential is defined by functions that are constructed from clamped cubic splines. The values of these splines at the nodes, along with their corresponding derivatives, are provided in Tables S1 to S5.

Table S1 – φ function values and their derivatives at the boundaries.

r (Å)	ϕ(eV)	ϕ'(eV/ Å)
1.8	0.94649	-3.89750
2.2	0.04520	-
2.6	-0.08618	-
3.0	-0.01267	-
3.4	0.00150	-
3.8	-0.01282	-
4.2	-0.01983	-
4.6	-0.01331	-
5.0	-0.00713	-
5.4	-0.00055	-
5.8	0.00000	0.00000

Table S2 – ρ function values and their derivatives at the boundaries.

r (Å)	ρ	ρ'(1/ Å)
1.8	0.28577	-0.422776
2.2	0.13015	-
2.6	0.07385	-
3.0	0.05298	-
3.4	0.02020	-
3.8	0.00435	-
4.2	-0.00243	-
4.6	-0.00137	-
5.0	-0.00074	-
5.4	-0.00269	-
5.8	0.00000	0.00000

Table S3– F function values and their derivatives at the boundaries.

$\overline{\rho}$	F(eV)	F'(eV)
0.0	0.00000	-4.92948
0.4	-2.23926	-
0.8	-3.45328	-
1.2	-3.50072	-
1.6	-3.32309	0.00000

Table S4 – u function values and their derivatives at the boundaries.

r (Å)	u($\frac{\sqrt{eV}}{Å}$)	u'($\frac{\sqrt{eV}}{Å^2}$)
1.8	-0.43626	1.22434
2.2	-0.13783	-
2.6	-0.07757	-
3.0	-0.03992	-
3.4	-0.00635	-
3.8	-0.00264	-
4.2	-0.00154	-
4.6	-0.00189	-
5.0	-0.00066	-
5.4	-0.00095	-
5.8	0.00000	0.00000

Table S5 – w function values and their derivatives at the boundaries.

r (Å)	w($\frac{\sqrt{eV}}{Å^2}$)	w'($\frac{\sqrt{eV}}{Å^3}$)
1.8	0.28976	-0.80558
2.2	0.08355	-
2.6	0.02457	-
3.0	0.00796	-
3.4	0.00033	-
3.8	-0.00052	-
4.2	0.00036	-
4.6	0.00032	-
5.0	-0.00026	-
5.4	-0.00000	-
5.8	0.00000	0.00000

3. Methodology for Potential Characterization

- Crystal Structure & Elasticity

To determine the lattice constant (*a* for FCC; *a* and *c* for HCP), bulk modulus (B), and elastic constants (C_{ij}), a periodic simulation cell containing 2048 atoms (8×8×8 unit cells) was first relaxed at 0 K. Subsequently, the system was equilibrated in the isothermal–isobaric (NPT) ensemble for 100 ps at the target temperature, matching the experimental conditions used for reference data. The lattice constant was obtained by averaging the instantaneous cell dimensions over this equilibration period. Once thermal and mechanical equilibrium were established, the bulk modulus and elastic constants C_{ij} were computed using the stress–strain approach. Incremental deformations were applied, with ±0.1% for the elastic constants and ±1.0% for the bulk modulus. For each deformation, the system was equilibrated in the canonical (NVT) ensemble at the target temperature, and the resulting stress tensors were averaged over a 100 ps time window.

- Cohesive Energy & Relative Phase Stability

The cohesive energy per atom (E_c) was obtained from the same relaxed configuration at 0 K, by dividing the total potential energy of the system by the number of atoms in the simulation cell.

The 0 K enthalpy difference between HCP and FCC crystal phases was estimated from relaxed per-atom potential energies. Periodic supercells of each phase were constructed and fully relaxed to zero external pressure at 0 K. After relaxation, the potential energy per atom (E_{hcp} and E_{fcc}) was extracted, which at zero temperature and pressure approximates the enthalpy per atom. The enthalpy difference was computed as

$$\Delta H^{fcc\rightarrow hcp} = H_{hcp} - H_{fcc} \approx E_{hcp} - E_{fcc}$$

- Interfaces & Stacking Faults

Surface energies were determined by comparing the total energies of relaxed and equilibrated bulk and slab configurations for each crystallographic orientation. Slab models were constructed to expose two equivalent free surfaces: (0001) for hcp

structures, and (100), (110), and (111) for fcc structures. A vacuum region was introduced along the surface normal to prevent interactions between periodic images. Both bulk and slab systems were equilibrated at 5 K under identical conditions. The surface energy was computed as

$$\gamma_s = \frac{E_{slab} - E_{bulk}}{2A}$$

where E_{slab} and E_{bulk} are the average total energies of the slab and bulk systems, respectively, and A is the surface area. Energies were averaged over 20 ps.

The intrinsic stacking-fault energy (γ_{I_2}) of hcp cobalt was obtained by computing the generalized stacking-fault energy surface on the basal (0001) plane. A vacuum region was introduced along the out-of-plane direction, above and below the crystal, to prevent interaction between the top and bottom halves of the system after the fault displacement. A fully relaxed hcp supercell at zero pressure served as the reference configuration with energy E_0. Stacking faults were introduced by rigidly shifting the upper half of the crystal by in-plane displacements (Δx, Δy), while allowing the interplanar spacing to relax. After energy minimization, the excess energy per unit area was calculated as

$$\gamma(\Delta x, \Delta y) = \frac{E_{fault} - E_0}{A}$$

where A is the fault-plane area. The γ-surface was scanned and refined near the minimum, and the lowest energy along the I_2 path was reported as γ_{I_2}.

- Point Defects & Vacancy Diffusion

Vacancy formation energies (E_v^f) were calculated using 5×5×5 supercells relaxed to zero pressure at 0 K. After computing the total energy of the perfect structure E_0, one atom was removed, and the defective configuration was re-relaxed to obtain E_{defect}. The formation energy was evaluated as

$$E_v^f = E_{defect} - E_0 \frac{N-1}{N}$$

where N is the number of atoms in the perfect supercell.

The vacancy migration energy (E_v^m) was computed using nudged elastic band (NEB) calculations at 0 K. Supercells were fully relaxed at zero pressure, and a monovacancy was introduced to define the migration path between adjacent lattice sites. The NEB method was applied to obtain the minimum-energy path, and the migration barrier was taken as the energy difference between the saddle point and the initial configuration.

- Phase Transitions & High-Temperature Thermodynamics

The melting temperature of fcc cobalt was estimated using the two-phase coexistence method. A periodic simulation cell containing 5120 atoms and measuring approximately 28.0 × 28.0 × 70.0 Å³ was constructed and divided along the z-axis into two equal regions representing solid and liquid phases. After relaxation to zero external pressure, a stable solid–liquid interface was prepared by independently thermalizing the two halves. This configuration was then used as the initial condition for a series of simulations at different target temperatures under isotropic NPT conditions.

Each simulation was run at constant pressure with a 1 fs time step. The melting point was identified as the temperature at which the solid–liquid interface remained stationary, indicating phase coexistence. Thermodynamic quantities such as temperature, pressure, enthalpy, and volume were monitored to ensure equilibrium behavior.

To estimate the hcp–fcc phase transition temperature of cobalt ($T^{hcp \rightarrow fcc}$) we adopted a thermodynamic integration approach similar to that described by G. Pun et al.[1]. Molecular dynamics simulations were performed for independent hcp and fcc supercells equilibrated under zero pressure in the NPT ensemble across a range of temperatures. The enthalpy difference between phases was fitted to a quadratic form and integrated using the Gibbs–Helmholtz relation to obtain the free energy difference ΔG(T). The integration constant was fixed using harmonic free energy calculations at a low reference temperature using Phonopy software package[2]. The transition temperature was identified as the point where ΔG(T)=0.

- Phonons & Thermal Properties of the Solid

The lattice heat capacity at constant volume (C_V) was calculated within the harmonic approximation using Phonopy[2].

Phonon dispersion relations were computed within the harmonic approximation using Phonopy[2], based on force constants obtained from finite displacements.

The linear thermal expansion was determined from the equilibrium lattice dimensions at different temperatures, using the lattice parameter at 300K as reference. For each target temperature, a 864-atom supercell (6×6×6 unit cells) was first brought to the desired temperature using NPT dynamics over 50 ps. The lattice parameter was then obtained by averaging the instantaneous cell dimensions over the subsequent 50 ps, also under NPT conditions.

- Liquid State: Transport & Volumetric Properties

The shear viscosity of liquid cobalt was computed using the Green–Kubo formalism based on equilibrium molecular dynamics. A periodic simulation cell containing 16,384 atoms was equilibrated at the target temperature in the liquid state using an NPT protocol, followed by a switch to the NVT ensemble for data collection under stationary conditions. The viscosity was obtained from the time integral of the stress autocorrelation function (SACF) of the off-diagonal components of the pressure tensor, averaged over the three independent shear directions.

The SACF was also examined to ensure that the chosen correlation length was sufficient to capture the full decay of the signal. Time integration was performed using the trapezoidal rule. The total production run spanned 1 ns.

The density of liquid cobalt was evaluated along a cooling trajectory using NPT molecular dynamics at ambient pressure. A periodic simulation cell containing 365 atoms was first equilibrated in the liquid state at 2500 K, then cooled in 200 K steps down to 1600 K. At each temperature, the density was computed from the time-averaged volume over a 50 ps NPT simulation. The wall time of these simulations was also recorded to estimate the computational cost of each potential.

4. Numerical Stability and Energy Conservation

To validate the choice of the time step (Δt = 1fs) and ensure the absence of significant integration errors, we assessed the energy conservation of the system in the microcanonical ensemble. A bulk HCP supercell containing 864 atoms was first equilibrated at 300 K and subsequently evolved without a thermostat.

Figure S1 displays the fluctuation of the total energy over the course of the simulation. The data indicates a negligible energy drift of approximately 2.3×10^{-5} % per nanosecond. This high degree of energy conservation confirms the numerical stability of the angular-dependent potential using the selected integration parameters.

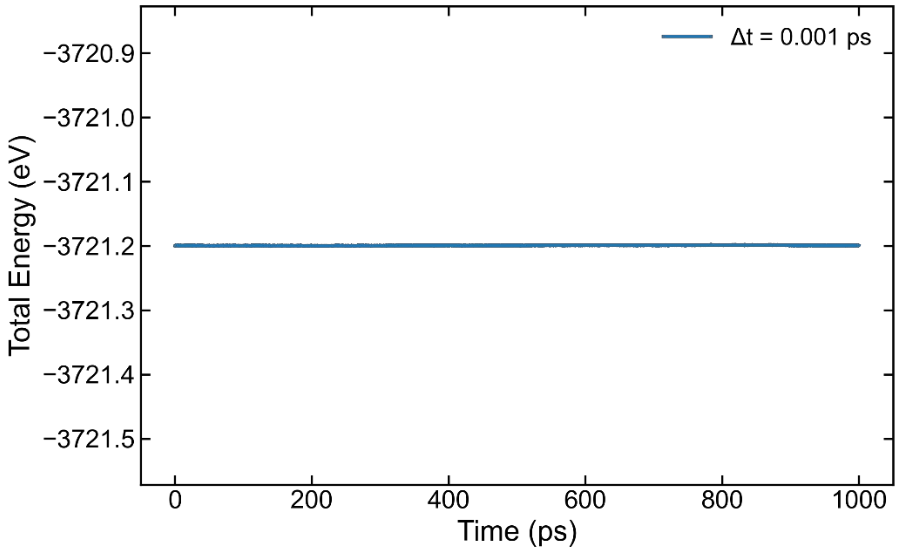

Figure S1. Evolution of the total energy for an 864-atom HCP Cobalt supercell simulated in the microcanonical (NVE) ensemble at an initial temperature of 300 K.

References

(1) Pun, G. P. P.; Mishin, Y. Embedded-Atom Potential for Hcp and Fcc Cobalt. *Phys Rev B Condens Matter Mater Phys* 2012, *86* (13). https://doi.org/10.1103/PhysRevB.86.134116.

(2) Togo, A.; Chaput, L.; Tadano, T.; Tanaka, I. Implementation Strategies in Phonopy and Phono3py. *Journal of Physics Condensed Matter*. Institute of Physics September 4, 2023. https://doi.org/10.1088/1361-648X/acd831.
